# *A* New Benzopyranyl Cadenane Sesquiterpene and Other Antiplasmodial and Cytotoxic Metabolites from *Cleistochlamys kirkii*

**DOI:** 10.3390/molecules24152746

**Published:** 2019-07-29

**Authors:** Stephen S. Nyandoro, Gasper Maeda, Joan J.E. Munissi, Amra Gruhonjic, Paul A. Fitzpatrick, Sofia Lindblad, Sandra Duffy, Jerry Pelletier, Fangfang Pan, Rakesh Puttreddy, Vicky M. Avery, Máté Erdélyi

**Affiliations:** 1 Chemistry Department, College of Natural and Applied Sciences, University of Dar es Salaam, Dar es Salaam P.O. Box 35061, Tanzania; 2 Department of Chemistry and Molecular Biology, University of Gothenburg, SE-412 96 Gothenburg, Sweden; 3 Sahlgrenska Cancer Centre, University of Gothenburg, SE-405 30 Gothenburg, Sweden; 4 Department of Chemistry-BMC, Uppsala University, SE-751 23 Uppsala, Sweden; 5 Discovery Biology, Griffith Institute for Drug Discovery, Griffith University, Nathan Q1d 4111, Australia; 6 Department of Biochemistry, McGill University, Montréal, QC H3G 1Y6, Canada; 7 Key Laboratory of Pesticide & Chemical Biology of Ministry of Education, Hubei International Scientific and Technological Cooperation Base of Pesticide and Green Synthesis, College of Chemistry, Central China Normal University, Luoyu Road 152, Wuhan 430079, China; 8 Department of Chemistry, University of Jyvaskyla, P.O. Box 35, FI-40014 Jyvaskyla, Finland

**Keywords:** *Cleistochlamys kirkii*, Annonaceae, benzopyranyl sesquiterpene, cleistonol, antiplasmodial activity, malaria, cytotoxicity

## Abstract

Phytochemical investigations of ethanol root bark and stem bark extracts of *Cleistochlamys kirkii* (Benth.) Oliv. (Annonaceae) yielded a new benzopyranyl cadinane-type sesquiterpene (cleistonol, **1**) alongside 12 known compounds (**2**–**13**). The structures of the isolated compounds were established from NMR spectroscopic and mass spectrometric analyses. Structures of compounds **5** and **10** were further confirmed by single crystal X-ray crystallographic analyses, which also established their absolute stereochemical configuration. The ethanolic crude extract of *C. kirkii* root bark gave 72% inhibition against the chloroquine-sensitive 3D7-strain malaria parasite *Plasmodium falciparum* at 0.01 μg/mL. The isolated metabolites dichamanetin, (*E*)-acetylmelodorinol, and cleistenolide showed IC_50_ = 9.3, 7.6 and 15.2 μM, respectively, against *P. falciparum* 3D7. Both the crude extract and the isolated compounds exhibited cytotoxicity against the triple-negative, aggressive breast cancer cell line, MDA-MB-231, with IC_50_ = 42.0 μg/mL (crude extract) and 9.6–30.7 μM (isolated compounds). Our findings demonstrate the potential applicability of *C. kirkii* as a source of antimalarial and anticancer agents.

## 1. Introduction

*Cleistochlamys* is a monotypic genus of the family, Annonaceae. It has a restricted distribution, being found only in some parts of Southern Africa. In Tanzania, *Cleistochlamys kirkii* (Benth.) Oliv. is found in Weme Forest Reserve, in the Rufiji flood plains, Rufiji District [1] and at Mchakama village, Kilwa District in Lindi Region [2]. *C. kirkii* is traditionally used in Mozambique to treat rheumatism, tuberculosis and haemorrhoid wounds [2,3,4,5]. Currently, there is no International Union of Conservation of Nature (IUCN) assessment recorded for *C. kirkii*; however, forest clearance for agriculture, settlement and timber logs for exports are posing a need for establishment of conservation measures [4]. Previous phytochemical investigations of its fruits, leaves and stem bark extracts revealed the presence of various antimicrobial and cytotoxic polyoxygenated heptenolides and cyclohexene derivatives [3]. We recently reinvestigated the leaves of *C. kirkii*, revealing new polyoxygenated cyclohexene derivatives and other constituents, some with activities against *Plasmodium falciparum* and human breast cancer cells (MDA-MB-231) [3]. These findings inspired the reinvestigation of the root and stem bark extracts of *C. kirkii* for their antiplasmodial and cytotoxic constituents, the results of which are reported in this paper.

## 2. Results and Discussion

### 2.1. Isolation and Identification of Compounds

**Figure 1:**
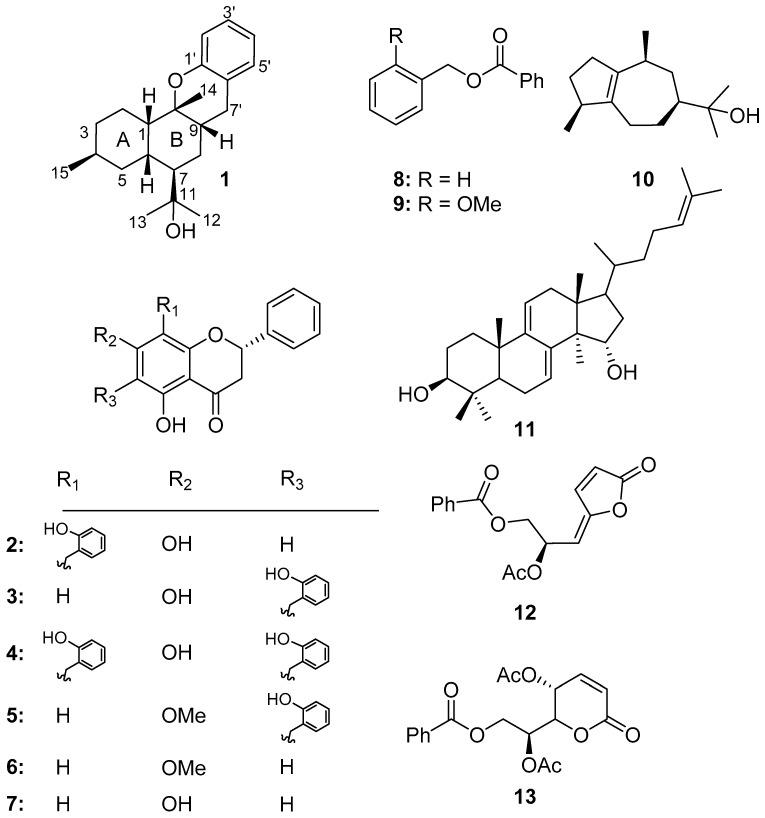
Chemical structures of compounds **1**–**13** isolated from *Cleistochlamys kirkii*.

Repeated silica gel chromatography followed by gel filtration with Sephadex^®^ LH-20 and/or further purification with HPLC gave a new benzopyranyl cadinane-type sesquiterpenoid (**1**) and twelve known secondary metabolites, **2**–**13** (Figure 1) from the root bark ethanolic extracts of *C. kirkii*. Compounds **2**–**4**, **7** and **11**–**13** (Figure 1) were also isolated from the stem bark ethanolic extracts of the same plant through chromatographic separation. The structures of the isolated compounds were established using UV, IR, CD, OR and NMR spectroscopic, and mass spectrometric techniques.

**Figure 2:**
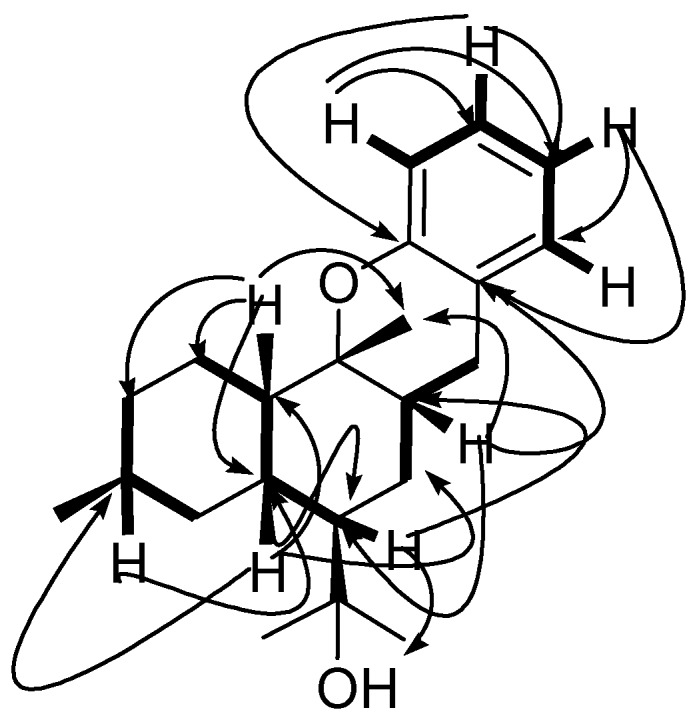
Some key COSY (bold lines) and HMBC (arrows) correlations for cleistonol (**1**).

Compound **1** {[α${]}_{D}^{20}$ −4.6 (c, 1.1, MeOH); CD (MeOH, λ_nm_ (∆ԑ; M^−1^ cm^−1^): (−4.73)_297_; (−5.4)_260_; (+3.78)_209_} was isolated as a white powder. Its molecular formula was determined to be C_22_H_32_O_2_, based on the HRESIMS (Supplementary Material Figure S8) data ([M + H]^+^ *m/z* 329.2467, calcd 329.2481). Its UV spectrum (MeOH) showed absorption at 275 nm, corresponding to an aromatic ring system. The IR spectrum showed O–H (3429 cm^−1^) and C=C (1640 cm^−1^) absorption bands, but lacked absorptions typical of other functional groups. The seven degrees of unsaturation (Index of Hydrogen Deficiency, IHD) suggested it was polycyclic. Its ^13^C-NMR spectrum (Table 1, Supplementary Material Figure S3) showed 22 peaks, which with help of a multiplicity edited HSQC (Supplementary Material Figure S4), were sorted into nine methines (four of which were in aromatic region), five methylenes, four methyls and four quaternary (two in the aromatic region) carbons. The ^1^H-NMR spectrum of **1** consisted of four aromatic signals at δ_H_ 6.75, 7.07, 6.81 and 7.04 ppm (Table 1, Supplementary Material Figure S1) with a coupling pattern reminiscent of an *ortho*-disubstituted ring. Based on COSY and TOCSY spectra (Supplementary Materials Figures S2 and S6), they were assigned to C-2′, C-3′, C-4′ and C-5′. The HMBC correlations (Figure 2, Supplementary Material Figure S5) of the NMR signals at δ_H_ 1.22 (3H, s, δ_C_ 27.6) and 1.19 (3H, s, δ_C_ 26.6) to C-12 and C-13 indicated that they are attached to a carbinol carbon, which is characteristic of cadinane-type sesquiterpenes possessing an isopropanoyl unit [6,7,8,9]. These protons also exhibited ^2^*J* and ^3^*J* heteronuclear couplings to the carbinol carbon at δ_C_ 73.7 (C-11) and to the methine carbon at δ_C_ 46.4 (C-7), respectively. The methyl signals at δ_H_ 0.86 (3H, d, *J* = 7.0 Hz; δ_C_ 16.0) and 1.47 (3H, s; δ_C_ 24.6) were deduced to be attached to the C-4 tertiary and C-10 quaternary carbons, respectively, based on their HMBC crosspeaks. The cyclohexane ring protons of ring A were identified based on COSY and TOCSY spectra. Hence, the H-4 (δ_H_ 2.08) methine proton coupled to the diastereotopic protons δ 1.50 and 1.35 (H-3α and H-3β), and δ 1.75 and 1.15 (H-5α and H-5β). The former diastereotopic protons, in turn, coupled to another set of diastereotopic protons at δ 1.81 and 1.59 (H-2α and H-2β), which showed further correlations to the bridge-head methine proton at δ 2.50 (H-1, δ_C_ 43.6). H-1, in turn, coupled to the bridgehead methine H-6 (δ 2.13, δ_C_ 43.3), which showed couplings to H-5 (Figure 2). The proton H-6 showed further coupling to the methine proton at δ 1.54 (C-7), which in turn, coupled to the diastereotopic protons δ 1.91 and 1.64 (H-8α and H-8β), connecting rings A and B. The H-8 methylene protons coupled with the methine δ 2.27 (H-9, δ_C_ 40.3), which showed COSY crosspeaks to the diastereotopic protons at δ 2.91 and 2.54 (H-7α′ and H-7′β). These diastereotopic protons showed HMBC crosspeaks to the aromatic carbons 153.5 (C-1′), 129.3 (C-5′), 31.6 (C-8) and 81.4 (C-10), indicating their benzylic positions. The continuous coupling pattern described above is characteristic of a dicyclic sesquiterpenoyl moiety (Figure 2), and the seven degrees of unsaturation are explained by the benzene, the pyran and the two cyclohexane rings. The relative configuration of Compound **1** was established based on the NOESY (Supplementary Material, Figure S7) correlations between the H-1 and the H-6, and the H-6 and the H-9 protons, analogous to the *cis*-fused muurolane-type cadinane sesquiterpene [6,7,8,9]. The *cis*-fused ring linkage of the sesquiterpene and benzopyranyl units was revealed by the NOE between H-9 and H-14, the former also showing NOE crosspeaks to H-1 and H-7′β. Moreover, the H-7′α benzyl proton showed NOE to H-7, indicating their suprafacial orientation. This new compound, cleistonol, **1**, isolated from the root bark of *C. kirkii*, was therefore, characterized as [2-(3,12a-dimethyl-2,3,4,4a,5,6,6a,7,12a,12b-decahydro-1H-benzo[c]xanthen-5-yl)-propan-2-ol].

**Scheme 1:**
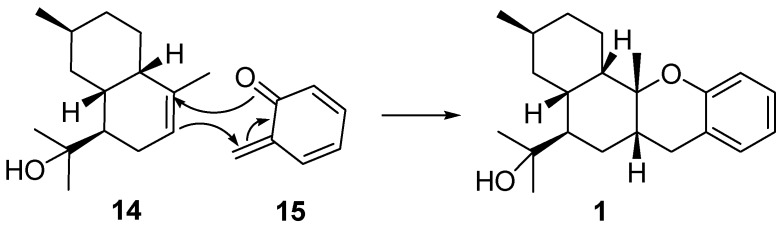
Proposed formation of benzopyran ring of clestonol (**1**) from precursors (**14** and **15**).

Compound **1** belongs to a novel, rare class of sesquiterpenoids constituting a benzopyranoyl moiety. These are uncommon in nature. Similar compounds have so far been reported only from a few members of the genus *Uvaria* (Annonaceae), namely *Uvaria angolensis* [10], *U. lucida* ssp. *lucida* [11,12] and *U. tanzaniae* [13]. Notwithstanding, this is the first report of a benzopyranyl cadinane-type sesquiterpene. The observation of such compounds from *Cleistochlamys* and *Uvaria* has a chemotaxonomic significance, as it may reveal their coevolution, yielding analogous anabolic enzyme systems. Similar to the previously reported benzopyranyl sesquiterpenes [10,11,12,13], clestonol (**1**) is envisioned to be formed via a Diels–Alder cycloaddition, involving the coupling of cadinenoyl sesquiterpene (**14**) with quinone methide (**15**) precursors leading to a benzopyran ring, as depicted in Scheme 1. The stereochemistry of the C9–C10 bond of **1**, confirmed by the NOE of H-9 and H-14, is in agreement with a concertic Diels–Alder reaction.

Besides cleistonol (**1**), twelve known compounds, chamanetin (**2**) [14,15], isochamanetin (**3**) [14,15], dichamanetin (**4**) [14,15], 7-methoxyisochamanetin (**5**) [16], pinostrobin (**6**) [17], pinocembrin (**7**) [3], benzylbenzoate (**8**) [18], 2-methoxybenzyl benzoate (**9**) [18], guaiol (**10**) [19], polycarpol (**11**) [3,15,20], (*E*)-acetylmelodorinol (**12**) [3], and cleistenolide (**13**) [3,15] were identified from the bark extract of *C. kirkii*, by comparison of their spectroscopic data with those reported in the literature.

**Figure 3:**
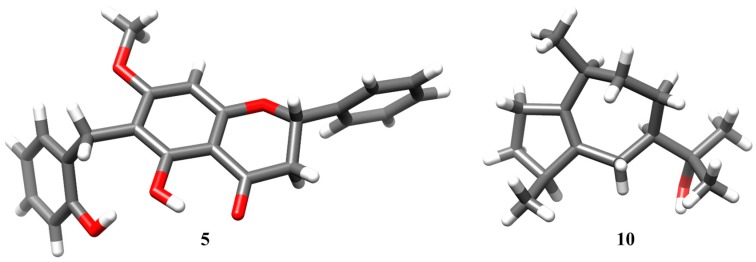
X-ray crystal structure of 7-methoxyisochamanetin (**5**) and guaiol (**10**).

The absolute configurations for 7-methoxyisochamanetin (**5**) and guaiol (**10**) were confirmed by using X-ray diffraction analysis (Figure 3). Both **5** and **10** crystallize in non-centrosymmetric space groups. The structure of **5** was solved in the orthorhombic space group *P*2_1_2_1_2_1_ and its absolute configuration was confirmed with a Flack Parameter of −0.07(5), whilst **10** was solved in the tetragonal space group *P*3_2_ with a Flack Parameter of 0.01(6). The levorotatory optical activity of Compound **5** is in good agreement with the *S*-configuration at C-2, determined by X-ray diffraction. The levorotatory optical rotation of the flavonoids **2**–**4**, **6** and **7** suggests that they also possess *S*-configuration at C-2 [21,22].

### 2.2. Antiplasmodial, Cytotoxic and Translation Inhibition Activities 

As part of our efforts to identify novel antimalarial and cytotoxic phytoconstituents from Tanzanian medicinal plants, we screened the crude extract of *C. kirkii* and its isolated constituents for their *in vitro* activity against the chloroquine-sensitive strain of *Plasmodium falciparum* (3D7), against the aggressive human breast cancer cell line MDA-MB-231, and for translational inhibitory activity, following previously established protocols [2,23,24]. We used Krebs-2 in vitro translation extracts programmed with a bicistronic firefly-HCV IRES-Renilla luciferase mRNA construct, to concurrently monitor both cap-dependent and cap-independent translation. The crude extract and some of its isolated constituents exhibited antiplasmodial activities, as shown in Table 2. The *C. kirkii* root bark crude extract (CKRE) showed 72% inhibition at 0.01 μg/mL against *P. falciparum* 3D7, indicating high effectiveness of its ingredients. Indeed, the pure compounds exhibited antiplasmodial activities of 62%–100% in the concentration range 10.0–40.0 μM. Compounds **12** and **4** were the most effective, with IC_50_ values of 7.6 and 9.30 μM, respectively (Table 2).

Comparing the antiplasmodial activities of the *C*-benzylated flavonoids **2**–**5** the hydroxybenzyl group appears to be important for bioactivity. Accordingly, dichamanetin (**4**), which has two hydroxybenzyl group moieties, was the most potent among the isolated compounds. As the hydroxybenzylated flavonoids were among the major constituents of the crude extract, this also showed high activity against *P. falciparum*. Our results corroborate the previous findings [15,25]. Heptenolide **13** isolated from the stem ethanolic extract along with flavonoids **2**–**4**, **7** and heptenolide **12** also exhibited potent antiplasmodial activity. Compound **12** showed comparable activity to its *cis* isomer that was isolated from the leaves of *C. kirkii* [2]. The new compound cleistonol (**1**) and Compounds **6**, **8**, **9**, **10** and **11** were either inactive or showed weak antiplasmodial activities at 40.0 μM. On the other hand, some of the tested compounds exhibited cytotoxicity with IC_50_ values 9.6–30.7 μM (Table 2), with dichamanetin (**4**) being the most active (IC_50_ 9.6 μM). The crude extract of *C. kirkii* exhibited cytotoxicity against the MDA-MB-231 cell line with an IC_50_ value of 42.0 μg/mL. Compounds **2**, **3**, **4, 7**, **12** and **13** showed very mild cytotoxicity at 20.0 μM when tested for translation inhibition activity in comparison to the positive control (Anisomycin). Some compounds were not tested for anticancer activity due to the limited amounts of samples available.

## 3. Materials and Methods

### 3.1. General Experimental Procedures

Analytical grade or distilled solvents, petroleum ether (boiling point 40–60 °C), ethyl acetate, methanol, *n*–hexane, acetone and *n*-pentane were used as received without further purification. Silica gel, 230–400 mesh ASTM particle size, was used for both vacuum liquid chromatography (VLC) and gravitational column chromatography (GCC) employing gradient elution. Further separation of semi-purified fractions was performed with Sephadex^®^ LH-20 (Pharmacia, Uppsala, Sweden) columns. The isolation process was monitored using thin layer chromatography (TLC), with the TLC plates being visualized with an SK-112005 TLC UV lamp (254 and 366 nm) (Scankemi, Täby, Sweden) and using anisaldehyde spraying reagent to observe UV-negative compounds, and the color changes of UV positive compounds. The anisaldehyde spraying reagent was prepared by mixing 2.0 mL of *p*-methoxybenzaldehyde, 5.0 mL of concentrated sulfuric acid, 10.0 mL of glacial acetic acid, and 80.0 mL of absolute ethanol or methanol. Melting points were determined on a Buchi B-545 melting point apparatus (Büchi Labortechnik AG, Flawil, Schwitzerland). An FT-IR spectrometer (Perkin-Elmer, Waltham, MA, USA) MIR 450–4000 cm^−1^ with 1.0 cm^−1^ resolution was used to record the IR spectra. The UV measurements were performed on a Cary 100 UV-VIS spectrophotometer (Varian, Inc., Palo Alto, California, USA). The optical rotation (OR) and circular dichroism (CD) spectra were acquired using a 341LC OROT polarimeter (Perkin-Elmer, Waltham, MA, USA) at a wavelength of 589 nm and a temperature of 20.0 °C, and a JASCO J-715 spectrometer (Jasco, Corp., Tokyo, Japan), respectively. LC–MS chromatograms were recorded on a Perkin-Elmer PE SCIEX 150 EX instrument (Perkin Elmer, Waltham, MA, USA) utilizing H_2_O/MeCN 80:20–20:80 gradient solvent systems with 0.2% HCO_2_H. High-resolution mass spectra (HRMS) were acquired on a Q-TOF-LC/MS spectrometer (Micromass, Wythenshawe, Waters Inc., Manchester, UK) utilizing a H_2_O/MeCN gradient solvent system by the company Stenhagen Analys AB, Gothenburg, Sweden. Further purifications of isolated compounds were carried out using preparative HPLC with a Waters 600E system (Waters Corp, Milford, MA, USA), with an H_2_O/MeCN or H_2_O/MeOH gradient solvent system and the software Chromulan. NMR experiments were recorded on a Bruker Avance III HD 800 MHz (Bruker BioSpin AG, Fällanden, Switzerland), Varian VNMR-S 500 and Varian MR 400 spectrometers (Agilent, Palo Alto, California, USA), utilizing deuterated methanol and chloroform as solvents. Spectra were processed using the software MestreNova (version12.0, Mestrelab Research, S.L., Santiago de Compostela, Spain).

### 3.2. Plant Materials

The root and stem barks of *C. kirkii* were collected on 19 March, 2013 from Mchakama village, part of Kilwa District in Lindi Region, Tanzania by S.S.N. under the guidance of Mr. Yahaya S. Abeid, a taxonomist at the Herbarium of the Botany, University of Dar es Salaam. The plant was identified in the field authenticated at the Herbarium, Botany Department of the University of Dar es Salaam, where a voucher specimen is deposited with the reference number YSA 3652.

### 3.3. Extraction and Isolation of Compounds

The root bark of *C. kirkii* was dried in air for two weeks, then ground to powder (1.14 kg). The ground materials were soaked twice and consecutively in ethanol for 48 h, filtered and concentrated using a rotary evaporator at 40 °C, to afford 121 g crude extract. The *C. kirkii* root bark ethanol extract (115 g) was subjected to vacuum liquid chromatography (VLC) using a solvent gradient from 20% EtOAc/*i*-hexane to 100% EtOAc then 10% methanol/EtOAc to afford six fractions of ca. 800 mL each, based on TLC analysis. The fraction obtained at 20% EtOAc/*i*–hexane was subjected to gravitational column chromatography (GCC) with a gradient elution from 100% *i*-hexane–25% EtOAc/*i*-hexane to obtain 15 fractions of approximately 150 mL each. Fractions 11–12 were combined based on TLC analysis and further purified by HPLC using 50:50 and 70:30 H_2_O/MeOH solvent systems to yield benzylbenzoate (**8**, 6.6 mg) and 2-methoxybenzylbenzoate (**9**, 4.7 mg). Fraction 3, obtained at 40% using VLC, was subjected to GCC and eluted slowly using 10%–50% EtOAc/*i*–hexane solvent system affording 60 fractions of ca 25.0 mL each. Subfraction 5 of Fraction 3 was crystallized with 10% EtOAc/*i*–hexane solvent system to yield polycarpol (**11**, 37.0 mg), while Subfraction 7 was subjected to HPLC with 70:30 H_2_O/MeOH eluent to yield pinostrobin (**6**, 4.6 mg). Subfractions 18–21 from Fraction 3 were combined and separated by HPLC to yield 7-methoxyisochamanetin (**5**, 2.8 mg) and cleistonol (**1**, 6.2 mg). Subfraction 27 was purified by HPLC and crystallized at room temperature in 70:30 H_2_O/MeOH to give guaiol (**10**, 8.2 mg). Subfraction 28 from the mother column was purified by HPLC using 40:60 H_2_O/MeOH to yield isochamanetin (**3**, 7.3 mg). Furthermore, Subfractions 32 and 33 were combined based on TLC analysis and purified by HPLC using a 50:50 H_2_O/MeOH solvent system to give chamanetin (**2**, 7.1 mg), dichamanetin (**4**, 12.0 mg), (*E*)-acetylmelodorinol (**12**, 1.4 mg) and cleistenolide (**13**, 2.4 mg).

The air-dried and pulverized stem bark of *C. kirkii* was soaked twice in EtOH at room temperature for 48 h, yielding 43 g of crude extract following evaporation of the solvent. The extract was adsorbed on silica gel, loaded on a silica gel column, and eluted with 10%–100% EtOAc in *i*-hexane and subsequently with 5%–20% MeOH in EtOAc, forming 8 fractions based on TLC. Fraction 3 was eluted with 20%–40% EtOAc in *i*-hexane yielding pinocembrin (**7**, 18.0 mg), polycarpol (**11**, 9.0 mg) and *E*-acetylmelodorinol (**12**, 24.0 mg). Fractions 4–6 were combined and subjected to GCC eluting with 30%–75% EtOAc in *i*-hexane, yielding *E*-acetylmelodorinol (**12**, 6.0 mg), chamanetin (**2**, 36.0 mg), isochamanetin (**3**, 16.0 mg) and dichamanetin (**4**, 54.0 mg).

### 3.4. Antiplasmodial Asexual Assay

Antiplasmodial activity was determined using an imaging-based assay method, as previously described by Duffy and Avery [26]. Initially, % inhibition was determined for all compounds, followed by determination of IC_50_ for the most active ones.

### 3.5. Cytotoxicity Assay

The crude extract and isolated constituents were evaluated against the MDA-MB-231, triple-negative, aggressive breast cancer cell line, as previously reported by Nyandoro et al [2].

### 3.6. Translation Inhibition Assay

Translation inhibition assays were performed following a previously established method [27].

## 4. Conclusions

Phytochemical investigation of the root and stem bark ethanol extracts of *C. kirkii* yielded the three *C*-benzylated flavonoids chamanetin (**2**), isochamanetin (**3**) and dichamanetin (**4**), the Anonnaceae chemotaxonomic marker triterpenenoid polycarpol (**11**), and the heptenolides *E*-acetylmelodorinol (**12**) and cleistenolide (**13**). Pinocembrin (**7**) was obtained from the stem bark extract only. The root bark extract has further provided the new benzopyranyl cadinane-type sesquiterpenoid, cleistonol (**1**), whose absolute configuration remains undetermined, and five known compounds (**5**, **6**, **8**, **9** and **10**). Isolation of the novel sesquiterpenoid, **1**, is of chemotaxonomic importance as it supports the phylogenetic relationship between the genera *Cleistochlamys* and *Uvaria*. 

The root bark crude extract of *C. kirkii* showed promising antiplasmodial (0.01 μg/mL, 70% inhibition) and anticancer activities (IC_50_ 42.0 μg/mL). Its isolated constituents dichamanetin (**4**), *E*-acetylmelodorinol (**12**) and cleistenolide (**13**) exhibited activity against *P. facliparum* (3D7) with IC_50_ 9.3, 7.6 and 15.2 μM, respectively. Compounds **2**, **3**, **4**, **7**, **12**, and **13** were active against MDA-MB-231 cells at IC_50_ 22.7, 11.6, 9.6, 13.2, 18.6 and 30.7 μM, respectively. This indicates the potential applicability of *C. kirkii* as a source of antimalarial, and even more likely of anticancer agents. None of the cytotoxic metabolites showed appreciable translation inhibitory activity, which was evaluated in an attempt to establish their mode of action.

## Figures and Tables

**Table 1:** ^1^H (800 MHz) and ^13^C (400 MHz) NMR spectroscopic data for cleistonol (**1**) acquired in CDCl_3_.

Position	δ_H_ (m, *J*, Hz)	δ_C_
1	2.50 (ddd, 10.7, 7.7, 2.1)	43.6
2α	1.81 (dddd, 12.8, 11.0, 10.9, 7.1)	26.5
2β	1.59 (m)	
3α	1.50 (dddd, 11,9, 11.6, 5.8, 2.0)	32.8
3β	1.35 (12.3, 7.3, 2.6, 2.6)	
4	2.08 (m)	39.0
5α	1.75 (ddd, 13.7, 3.6, 1.7)	29.0
5β	1.15 (ddd, 13.7, 10.2, 3.0)	
6	2.13 (dddd, 12.9, 10.7, 6.7, 3.8)	43.3
7	1.54 (m)	46.4
8α	1.91 (ddd, 14.3, 4.8, 2.4)	31.6
8β	1.64 (ddd, 14.3, 11.2, 3.8)	
9	2.27 (dddd, 12.3, 4.9, 4.6, 4.6)	40.3
10	-	81.4
11	-	73.9
12	1.22 (s)	27.6
13	1.19 (s)	26.6
14	1.47 (s)	24.6
15	0.86 (d, 7.2)	16.0
1′	-	153.5
2′	6.75 (d, 8.0)	117.3
3′	7.07 (dd, 8.0, 8.0)	127.3
4′	6.81(dd,8.0, 8.0)	119.7
5′	7.04 (d, 8.0)	129.3
6′	-	123.0
7′α	2.91 (dd, 16.4, 12.3)	28.4
7′β	2.54 (dd, 16.4, 4.9)	

**Table 2:** Antiplasmodial and cytotoxic activities (IC_50_ and % inhibition) of the selected constituents of *Cleistochlamys kirkii*.

Compound/Extract	3D7	MDA-MB-231
CKRE (crude)	72% ^c.^*	42.0 ^a^ (μg/mL)
Cleistonol (**1**)	IA ^b^	NT
Chamanetin (**2**)	62% ^b^	22.7 ^a^
Isochamanetin (**3**)	72% ^b^	11.6 ^a^
Dichamanetin (**4**)	9.3 ^a^	9.6 ^a^
7-Methoxyisochamanetin (**5**)	84% ^b^	NT
Pinostrobin (**6**)	IA ^b^	NT
Pinocembrin (**7**)	10% ^b^	30.7 ^a^
Benzylbenzoate (**8**)	25% ^b^	NT
2-Methoxybenzylbenzoate (**9**)	IA ^b^	NT
Guaiol (**10**)	IA ^b^	NT
Polycarpol (**11**)	6% ^b^	NT
(*E*)-Acetylmelodorinol (**12**)	7.6 ^a^	13.2 ^a^
Cleistenolide (**13**)	15.2 ^a^	18.6 ^a^
Lupeol (reference)		0.089
Artesunate (reference)	0.00048	

^a^ IC_50_. ^b^ Percentage growth inhibition at 40.0 μM. ^c^ At 0.01 μg/mL (* considering that the active compound is only 0.1% of the mass of crude). The inhibitory activities are given as the mean value of at least two independent measurements. IA—Inactive. NT—Not tested.

## Supplementary Materials

The Supplementary Materials are available online. Figure S1: The ^1^H-NMR Spectrum of cleistonol (**1**) Measured at 800 MHz and Acquired in CDCl_3_; Figure S2: The H/H COSY Spectrum of cleistonol (**1**) Measured at 800 MHz and Acquired in CDCl_3_; Figure S3: The ^13^C-NMR Spectrum of cleistonol (**1**) Measured at 800 MHz and Acquired in CDCl_3_; Figure S4: The HSQCED Spectrum of cleistonol (**1**) Measured at 800 MHz and Acquired in CDCl_3_; Figure S5: The HMBC Spectrum of cleistonol (**1**) Measured at 800 MHz and Acquired in CDCl_3_; Figure S6: The TOCSY Spectrum of cleistonol (**1**) Measured at 800 MHz and Acquired in CDCl_3_; Figure S7: The NOESY Spectrum of cleistonol (**1**) Measured at 800 MHz and Acquired in CDCl_3_; Figure S8: The HRESIMS of cleistonol (**1**). Spectroscopic data of compounds (**2**–**13**), and X-ray crystallographic data of **5** (CCDC 1937081) and **10** (CCDC 1937082). Original FIDs are available, open access, at Zenodo with DOI: 10.5281/zenodo.3271696.

## References

[1] Verdcourt B. (1971). Flora of tropical East Africa: Annonaceae.

[2] Nyandoro S.S., Munissi J.J., Gruhonjic A., Duffy S., Pan F., Puttreddy R., Holleran J.P., Fitzpatrick P.A., Pelletier J., Avery V.M. (2017). Polyoxygenated cyclohexenes and other constituents of *Cleistochlamys kirkii* leaves. J. Nat. Prod..

[3] Samwel S., Mdachi S.J.M., Nkunya M.H.H., Irungu B.N., Moshi M.J., Moulton B., Luisi B.S. (2007). Cleistenolide and cleistodienol: Novel bioactive constituents of *Cleistochlamys kirkii*. Nat. Prod. Commun..

[4] Nyandoro S.S. (2014). Some rare Tanzanian plant species as sources of less common metabolites: Biomedical potential and conservation status. J. Pharmacogn. Phytochem..

[5] Verzár R., Petri G. (1987). Medicinal plants in Mozambique and their popular use. J. Ethnopharm..

[6] Bordoloi M., Shukla V.S., Nath S.C., Sharma R.P. (1989). Naturally occurring cadinenes. Phytochemistry.

[7] Chyu C.F., Ke M.R., Chang Y.S., Chien S.C., Kuo Y.H. (2007). New Cadinane-type sesquiterpenes from the roots of Taiwania Cryptomerioides Hayata. Helv. Chim. Acta..

[8] Kuo Y.H., Chen C.H., Chien S.C., Lin Y.L. (2002). Five new cadinane-type sesquiterpenes from the heartwood of *Chamaecyparis obtusa* var. formosana. J. Nat. Prod..

[9] Kuo Y.H., Chyu C.F., Lin H.C. (2003). Cadinane-type sesquiterpenes from the roots of Taiwania cryptomerioides Hayata. Chem. Pharm. Bull..

[10] Muhammad I., Waterman P.G. (1988). Chemistry of the Annonaceae, Part XXVI. The Uvarisesquiterpenes, a novel type of benzylated sesquiterpene from Uvaria angolensis. J. Nat. Prod..

[11] Mgani Q. (2012). Uvarisesquiterpene D: A new benzopyranyl sesquiterpene isolated from *Uvaria Lucida* ssp. Lucida. Tanzan. J. Sci..

[12] Weenen H., Nkunya M.H., El-Fadl A.A., Harkema S., Zwanenburg B. (1990). Lucidene, a bis (benzopyranyl) sesquiterpene from *Uvaria lucida* ssp. lucida. J. Org. Chem..

[13] Weenen H., Nkunya M.H., Mgani Q.A., Posthumus M.A., Waibel R., Achenbach H. (1991). Tanzanene, a spiro benzopyranyl sesquiterpene from *Uvaria tanzaniae* Verdc. J. Org. Chem..

[14] Achenbach H., Höhn M., Waibel R., Nkunya M.H., Jonker S.A., Muhie S. (1997). Oxygenated pyrenes, their potential biosynthetic precursor and benzylated dihydroflavones from two African Uvaria species. Phytochemistry.

[15] Pereira F., Madureira A.M., Sancha S., Mulhovo S., Luo X., Duarte A., Ferreira M.J.U. (2016). Cleistochlamys kirkii chemical constituents: Antibacterial activity and synergistic effects against resistant *Staphylococcus aureus* strains. J. Ethnopharmacol..

[16] Lasswell W.L., Hufford C.D. (1977). Cytotoxic C-benzylated flavonoids from *Uvaria chamae*. J. Org. Chem..

[17] Ching A.Y.L., Wah T.S., Sukari M.A., Lian G.E.C., Rahmani M., Khalid K. (2007). Characterization of flavonoid derivatives from Boesenbergia rotunda (L.). Malays. J. Anal. Sci..

[18] Kodpinid M., Thebtaranonth C., Thebtaranonth Y. (1985). Benzyl benzoates and o-hydroxybenzyl flavanones from Uvaria ferruginea. Phytochemistry.

[19] Liu T., Wang C.J., Xie H.Q., Mu Q. (2013). Guaiol—A naturally occurring insecticidal sesquiterpene. Nat. Prod. Commun..

[20] Jung J., Pummangura S., Chaichantipyuth C., Patarapanich C., Fanwick P., Chang C.J., McLaughlin J. (1990). New bioactive heptenes from *Melodorum fruticosum* (Annonaceae). Tetrahedron.

[21] Slade D., Ferreira D., Marais J.P.J. (2005). Circular dichroism, a powerful tool for the assessment of absolute configuration of flavonoids. Phytochemistry.

[22] Antus S., Baitzgacs E., Kajtar J., Snatzke G., Tokes A.L. (1994). Circular-Dichroism and Absolute-Configuration of Azaflavanones and Thiaflavanones. Liebigs. Ann. Chem..

[23] Atilaw Y., Duffy S., Heydenreich M., Muiva-Mutisya L., Avery V.M., Erdelyi M., Yenesew A. (2017). Three chalconoids and a pterocarpene from the roots of *Tephrosia aequilata*. Molecules.

[24] Deyou T., Gumula I., Pang F., Gruhonjic A., Mumo M., Holleran J., Duffy S., Fitzpatrick P.A., Heydenreich M., Landberg G. (2015). Rotenoids, flavonoids, and chalcones from the root bark of *Millettia usaramensis*. J. Nat. Prod..

[25] Nkunya M.H.H. (2002). Natural Chemicals for Disease and Insect Management.

[26] Duffy S., Avery V.M. (2012). Development and optimization of a novel 384-well anti-malarial imaging assay validated for high-throughput screening. Am. J. Trop. Med. Hyg..

[27] Novac O., Guenier A.S., Pelletier J. (2004). Inhibitors of protein synthesis identified by a high throughput multiplexed translation screen. Nucleic. Acids. Res..

