# Supplementary material for: A New Benzopyranyl Cadenane Sesquiterpene and Other Antiplasmodial and Cytotoxic Metabolites from Cleistochlamys kirkii
Source: Molecules. 2019 Jul 29;24(15):2746. doi: 10.3390/molecules24152746 (PMC6695775; doi:10.3390/molecules24152746)
Supplement: Supplementary file 1 [file molecules-24-02746-s001.pdf]

Supporting Information

A New Benzopyranyl Cadenane Sesquiterpene and Other Antiplasmodial and Cytotoxic Metabolites from *Cleistochlamys kirkii*

Stephen S. Nyandoro[1,2,*], Gasper Maeda[1,2], Joan J.E. Munissi[1], Amra Gruhonjic[2], Paul A. Fitzpatrick[3], Sofia Lindblad[4], Sandra Duffy[5], Jerry Pelletier[6], Fangfang Pan[7,8], Rakesh Puttreddy[8], Vicky M. Avery[5], Máté Erdélyi[2,4*]

1 Chemistry Department, College of Natural and Applied Sciences, University of Dar es Salaam, P.O. Box. 35061, Dar es Salaam, Tanzania
2 Department of Chemistry and Molecular Biology, University of Gothenburg, SE-412 96 Gothenburg, Sweden
3 Sahlgrenska Cancer Centre, University of Gothenburg, Gothenburg, SE-405 30, Sweden
4 Department of Chemistry - BMC, Uppsala University, SE-751 23 Uppsala, Sweden
5 Discovery Biology, Griffith Institute for Drug Discovery, Griffith University, Nathan Q1d 4111, Australia
6 Department of Biochemistry, McGill University, Montréal, QC, H3G 1Y6, Canada
7 Key Laboratory of Pesticide & Chemical Biology of Ministry of Education, Hubei International Scientific and Technological Cooperation Base of Pesticide and Green Synthesis, College of Chemistry, Central China Normal University, Luoyu Road 152, Wuhan 430079 (P. R. China)
8 University of Jyvaskyla, Department of Chemistry, P.O. Box. 35, FI-40014, Jyvaskyla, University of Jyvaskyla, Finland

* Correspondence: nyandoro@udsm.ac.tz (S. S. N.); mate@kemi.uu.se (M. E.). Tel.: +255-754-206560 (S.S.N.); +46-72-9999166 (M.E.)

Table of Contents

1. NMR and Mass Spectra of Cleistonol (1) ……………………………………………………………S2

2. Spectroscopic Data of Known Compounds 2-13 ………………………………………………….....S6

3. X-Ray crystallography for 7-Methoxyisochamanetin (5) and Guaiol (10) ………………….……….S16

4. References…………………………………………………………………………………….……..S17

1. NMR and Mass Spectra of Cleistonol (1)

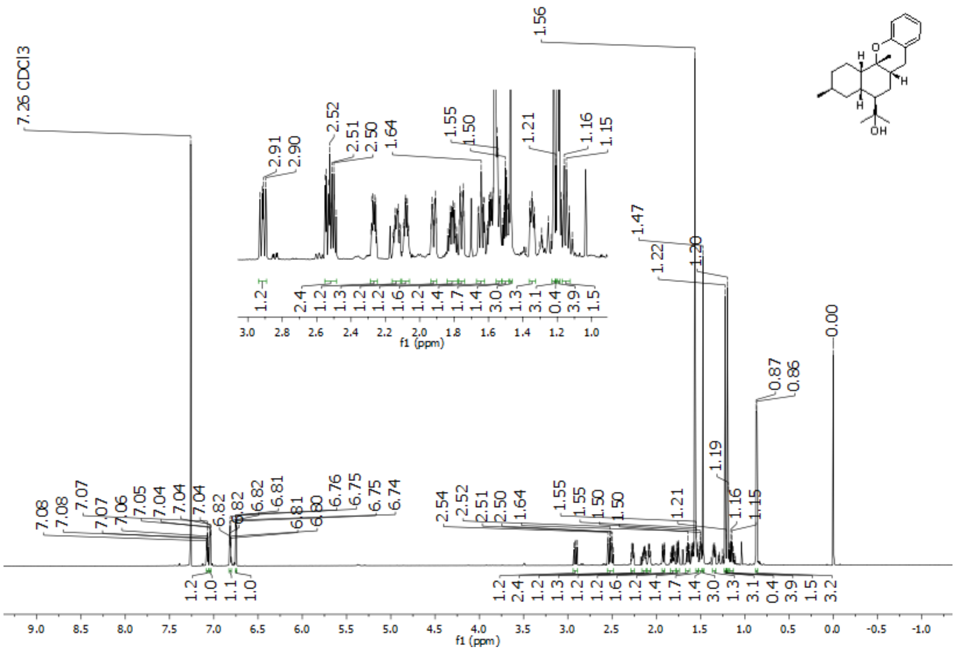

Figure S1. The ^{1}H NMR Spectrum of Cleistonol (**1**) Measured at 800 MHz and Acquired in $CDCl_3$.

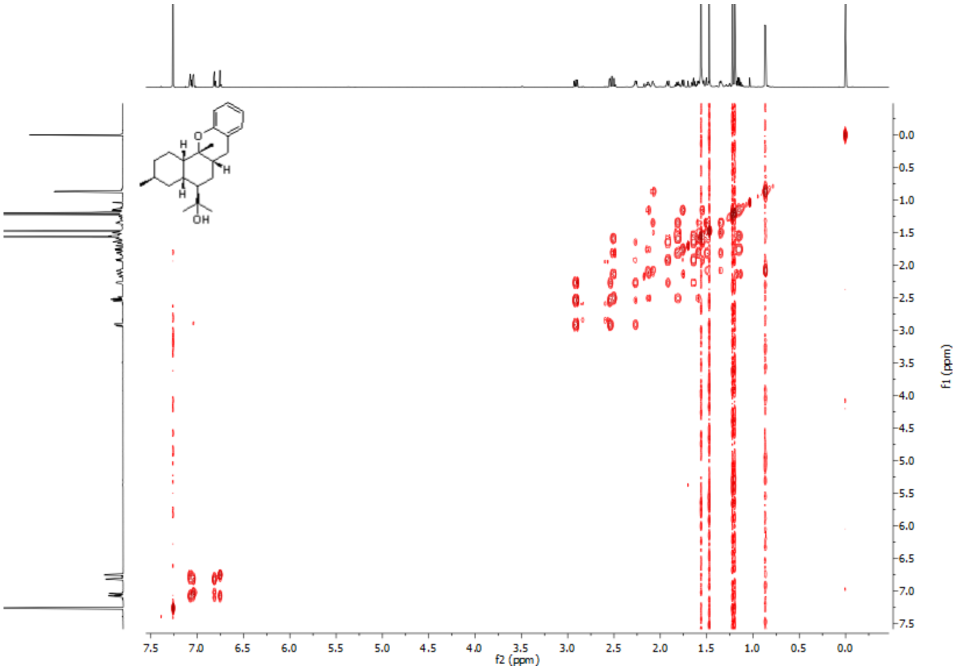

Figure S2. The H/H COSY Spectrum of Cleistonol (**1**) Measured at 800 MHz and Acquired in $CDCl_3$.

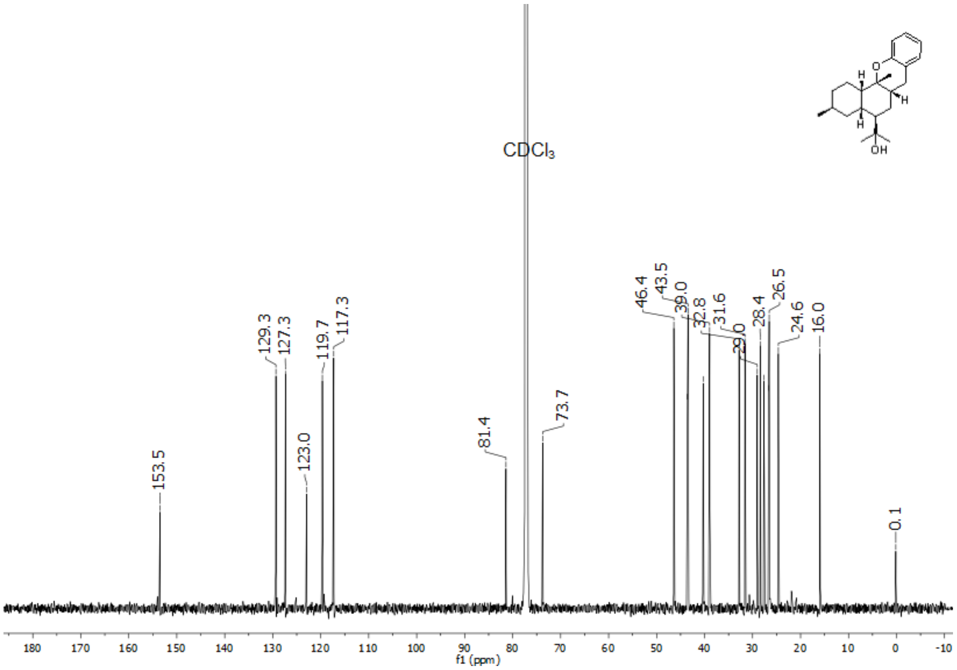

Figure S3. The ^{13}C NMR Spectrum of Cleistonol (**1**) Measured at 800 MHz and Acquired in $CDCl_3$.

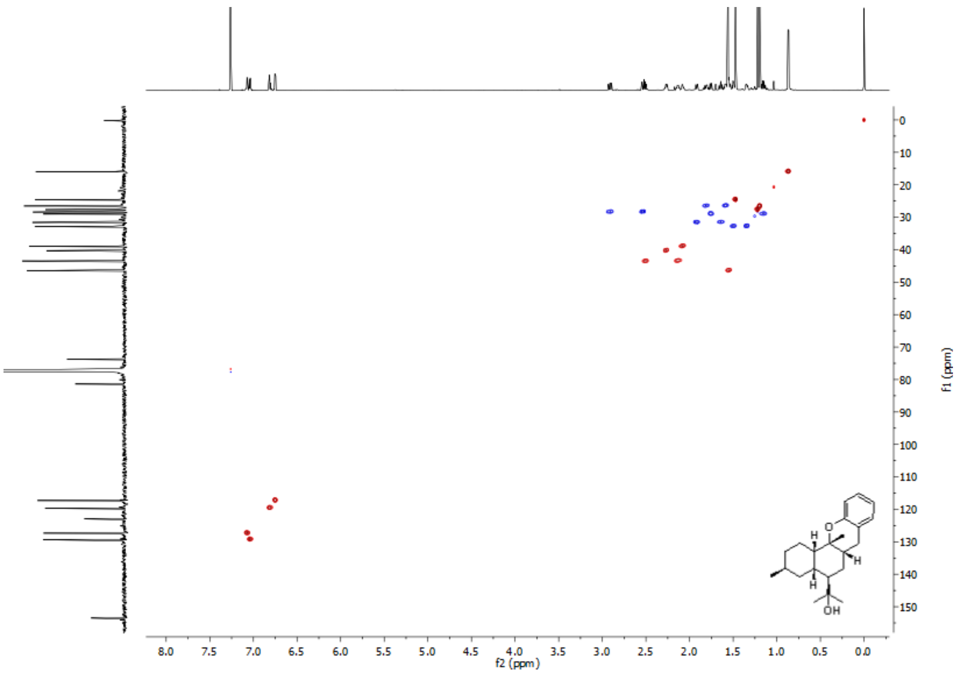

Figure S4. The HSQC Spectrum of Cleistonol (**1**) Measured at 800 MHz and Acquired in $CDCl_3$.

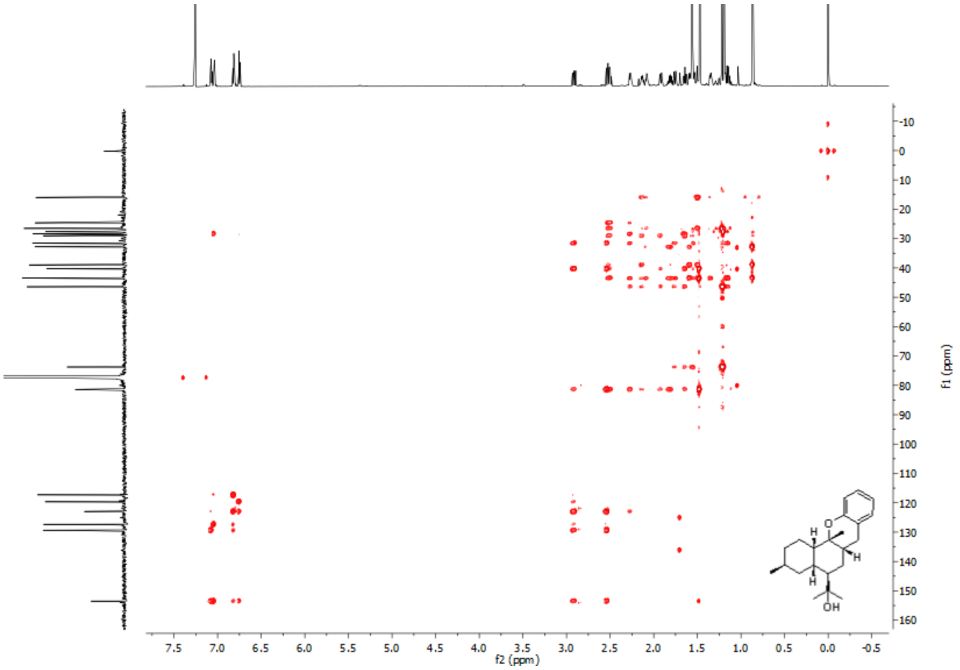

Figure S5. The HMBC Spectrum of Cleistonol (**1**) Measured at 800 MHz and Acquired in $CDCl_3$.

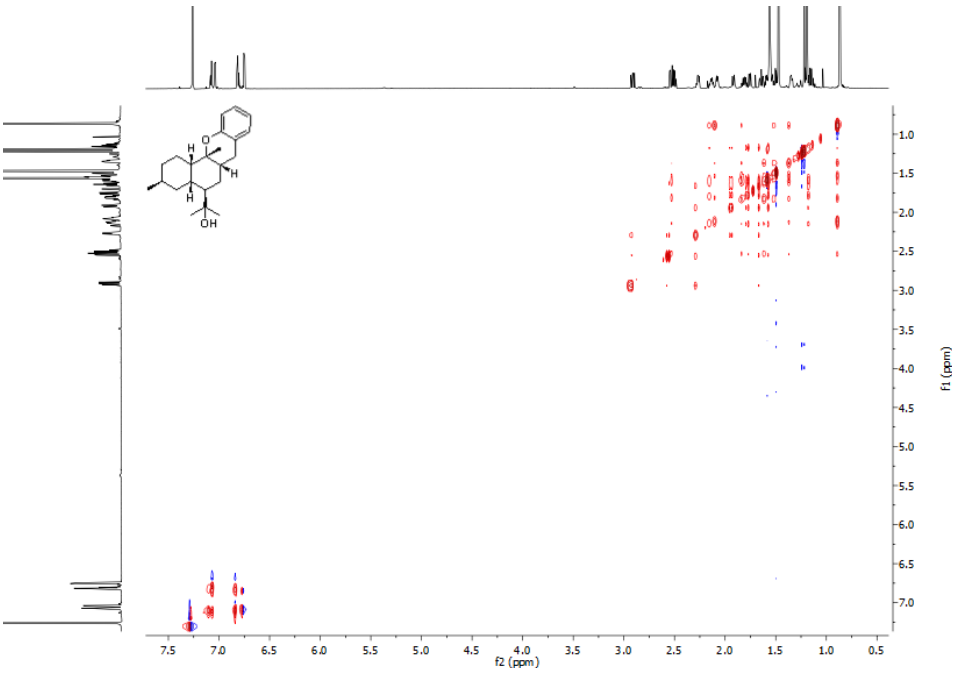

Figure S6. The TOCSY Spectrum of Cleistonol (**1**) Measured at 800 MHz and Acquired in $CDCl_3$.

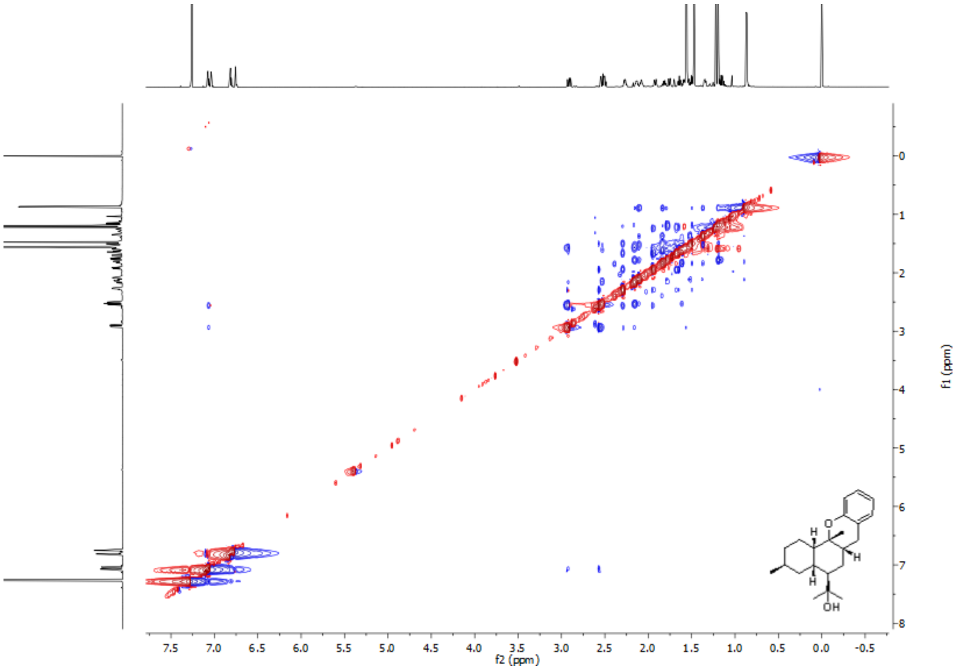

Figure S7. The NOESY Spectrum of Cleistonol (**1**) Measured at 800 MHz and Acquired in $CDCl_3$.

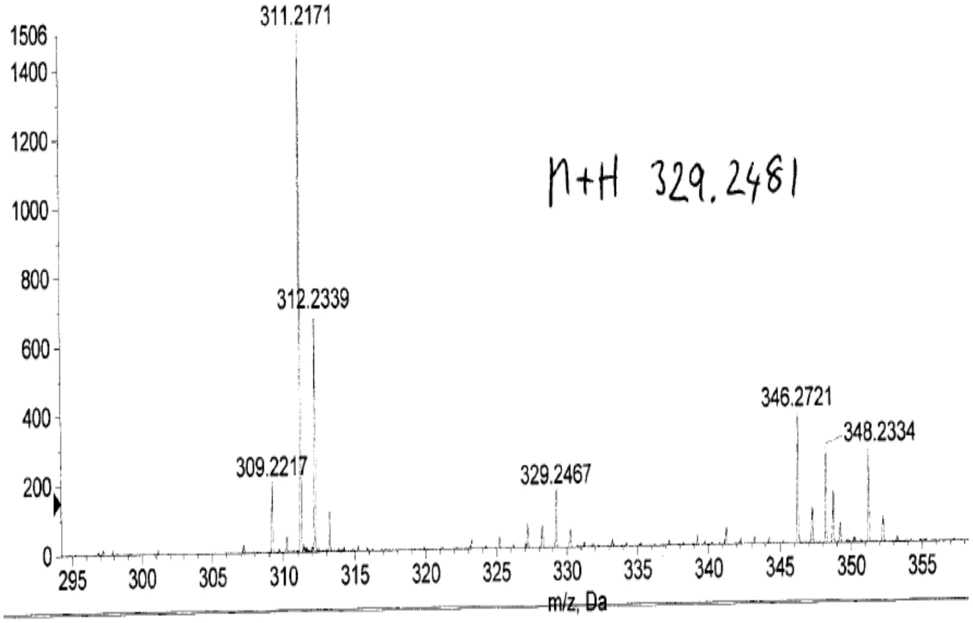

Figure S8. The HRESIMS of Cleistonol (**1**).

2. Spectroscopic Data of Known Compounds 2-13.

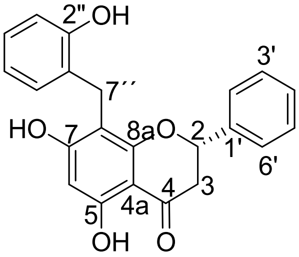

*Chamanetin (***2***)*: [1, 2] Yellow crystals. mp 200 - 204 °C; [$[\alpha]_D^{20}$ – 16.1 (c, 5.3, MeOH), UV (MeOH)$_{\lambda max}$ 290, 325 nm; CD (MeOH, λnm ($\Delta\varepsilon$; $M^{-1}cm^{-1}$): (- 7.67)$_{290}$; (+ 3.47)$_{216}$; IR (KBr) ν_{max} 3056, 1423, 1265, 895, 739 cm^{-1}; LC-MS *m/z* 363.5 $[M+H]^+$, 269.6, 257.5, 165.4, 149.6, 102.2, 74.1, 60.2; ^{1}H-NMR ($CDCl_3$, 25 °C) δ (800 MHz, ppm): 5.52 (1H, *dd*, *J* = 12.9, 3.1 Hz, H-2), 3.10 (1H, *dd*, *J* = 17.1, 12.9 Hz, H-3α), 2.81 (1H, *dd*, *J* = 17.1, 3.1 Hz, H-3β), 12.08 (1H, *s*, 5-OH), 6.03 (1H, *s*, H-6), 3.78 (2H, *s*, H-11), 7.39 - 7.50 (2H, *m*, H-2′/6′), 7.39 - 7.50 (2H, *m*, H-3′/5′), 7.50 (1H, *m*, H-4′), 6.79 (1H, *dd*, *J* = 8.0, 0.9 Hz, H-3″), 7.02, (1H, *m*, H-4″), 6.74 (1H, *td*, *J* = 7.5, 1.0 Hz, H-5″), 7.04 (1H, *dd*, *J* = 7.5, 1.6 Hz, H-6″); ^{13}C-NMR ($CDCl_3$, 25 °C) δ (200 MHz, ppm): 80.1 (C-2), 43.4 (C-3), 197.6 (C-4), 103.6 (C-4a), 163.3 (C-5), 97.0 (C-6), 164.7 (C-7), 107.3 (C-8), 161.3 (C-8a), 139.9 (C-1′), 127.3 (C-2′/6′), 129.7 (C-3′/5′), 129.6 (C-4′), 154.7 (C-2″), 115.9 (C-3″), 128.2 (C-4″), 121.1 (C-5″), 130.9 (C-6″), 22.9 (C-7″).

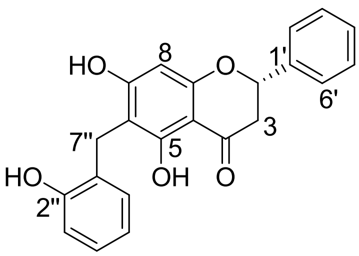

*Isochamanetin (**3**)*: [1, 2] Yellow powder. mp 212 - 214 °C; $[\alpha]_D^{20}$ - 4.3 (c, 0.7, MeOH), UV (MeOH)$_{\lambda max}$ 290, 335 nm; CD (MeOH, λnm (Δε; $M^{-1}cm^{-1}$): $(+9.23)_{331}$; $(- 40.16)_{288}$; $(+ 45.07)_{222}$; IR (KBr) ν_{max} 3418, 1638, 1265, 740 cm^{-1} ; LC-MS *m/z* 363.5 $[M+H]^+$, 271.6, 269.4, 259.6, 257.5, 241.3, 175.5, 165.3, 149.5, 147.0, 123.2, 107.4, 98.9, 81.1, 60.1; ^{1}H-NMR ($CDCl_3$, 25 ^{o}C) δ (800 MHz, ppm): 5.45 (1H, *dd*, *J* = 12.9, 3.0 Hz, H-2), 3.08 (1H, *dd*, *J* = 17.0, 13.0 Hz, H-3α), 2.76 (1H, *dd*, *J* = 17.0, 3.0 Hz, H-3β), 6.01 (1H, *s*, H-8), 3.83, 3.81 (2H, *q*, *J* = 3.9 Hz, H-7′′), 7.50 (2H, *d*, *J* = 7.3 Hz, H-2′/6′), 7.41 (2H, *t*, *J* = 7.7 Hz, H-3′/5′), 7.36 (1H, *m*, H-4′), 6.75 (1H, *dd*, *J* = 8.0, 1.0 Hz, H-3′′), 6.98 (1H, *dt*, *J* = 7.8, 1.6 Hz, H-4′′), 6.69 (1H, *dt*, *J* = 7.6, 1.2 Hz, H-5′′), 7.05 (1H, *dd*, *J* = 7.6, 1.2 Hz, H-6′); ^{13}C-NMR ($CDCl_3$, 25 ^{o}C) δ (200 MHz, ppm): 80.4 (C-2), 44.3 (C-3), 197.2 (C-4), 103.1 (C-4a), 162.8 (C-5), 109.0 (C-6), 162.8 (C-7), 96.3 (C-8), 155.7 (C-8a), 140.6 (C-1′), 127.3 (C-2′/6′), 129.7 (C-3′/5′), 129.6 (C-4′), 128.4 (C-1′′), 155.7 (C-2′′), 116.0 (C-3′′), 127.8 (C-4′′), 120.7 (C-5′′), 130.8 (C-6′′), 22.7 (C-7'').

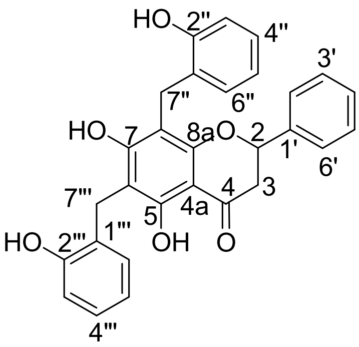

Dichamanetin (**4**): [1, 2] Yellow gum. $[\alpha]_D^{20}$ – 12.8 (c, 9.2, MeOH), UV (MeOH)λ_{max} 335, 295 nm; CD (MeOH, λnm ($\Delta\varepsilon$; $M^{-1}cm^{-1}$): (- 5.01)$_{380}$; (- 3.35)$_{253}$; (+ 1.11)$_{223}$; IR (KBr) ν_{max} 3054, 1630, 1420, 1265, 896, 739 cm^{-1}; LC-MS *m/z* 469.4 $[M+H]^+$, 429.1, 377.5, 375.5, 365.6, 363.5, 296.9, 281.5, 271.6, 269.6, 253.3, 213.3, 177.3, 149.5, 114.3; ^{1}H-NMR ($CDCl_3$, 25 ^{o}C) δ (800 MHz, ppm): 5.54 (1H, *dd*, *J* = 12.8, 3.0 Hz, H-2), 3.16 (1H, *dd*, *J* = 17.2, 12.8 Hz, H-3α), 2.86 (1H, *dd*, *J* = 17.2, 3.0 Hz, H-3β), 3.87 (2H, *d*, *J* = 6.9 Hz, H-7′′), 3.84 (2H, *s*, H-7′′′), 7.53 (2H, *d*, *J* = 7.3 Hz, H-2′/6′), 7.47 (2H, *t*, *J* = 7.5 Hz, H-3′/5′), 7.43 (1H, *m*, H-4′), 6.85 (1H, *dd*, *J* = 8.1, 0.9 Hz, H-3′′), 7.08 (1H, *m*, H-4′′), 6.82 (1H, *m*, H-5′′), 7.26 (1H, *dd*, *J* = 7.6, 1.3 Hz, H-6′′), 6.83 (1H, *m*, H-3′′′), 7.05 (1H, *m*, H-4′′′), 6.77 (1H, *m*, H-5′′′), 7.09 (1H, *m*, H-6′′′), 12.71 (1H, *s*, 5-OH), 8.16 (1H, *brs*, 7-OH), 8.16 (1H, *brs*, 2′′-OH), 8.16 (1H, *brs*, 2′′′-OH); ^{13}C-NMR ($CDCl_3$, 25 ^{o}C) δ (200 MHz, ppm): 79.9 (C-2), 43.0 (C-3), 197.6 (C-4), 103.2 (C-4a), 160.7 (C-5), 108.1 (C-6), 161.9 (C-7), 107.3 (C-8), 159.2 (C-8a), 139.5 (C-1′), 127.0 (C-2′/6′), 129.3 (C-4′), 127.2 (C-1′′), 154.0 (C-2′′), 115.7 (C-3′′), 128.0 (C-4′′), 121.1 (C-5′′), 131.1 (C-6′′), 22.3 (C-7"), 127.1 (C-1′′′), 153.8 (C-2′′′), 115.5 (C-3′′′), 128.0 (C-4′′′), 121.1 (C-5′′′), 130.9 (C-6′′′), 22.9 (C-7''').

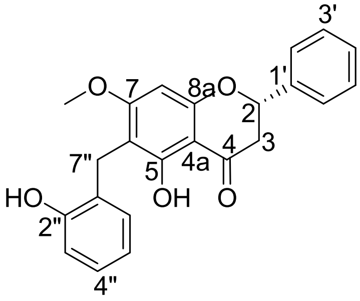

7-Methoxyisochamanetin (**5**): [3] White crystals. $[\alpha]_D^{20}$ – 5.6 (c, 2.1, MeOH), UV (MeOH)$_{\lambda max}$ 285, 330 nm; CD (MeOH, λnm (Δε; $M^{-1}cm^{-1}$): (+18.25)$_{334}$; (- 3.91)$_{302}$; (+ 2.49)$_{279}$; (- 8.41)$_{265;}$ IR (KBr) ν_{max} 3424, 1638, 1265, 739 cm^{-1}; LC-MS *m/z* 377.5 $[M+H]^+$, 318.3, 283.7, 271.7, 241.5, 205.2, 179.1, 167.5, 149.5, 125.2, 102.1; ^{1}H-NMR ($CDCl_3$, 25 ^oC) δ (800 MHz, ppm): 5.40 (1H, *dd*, *J* = 13.1, 3.1 Hz, H-2), 3.09 (1H, *dd*, *J* = 17.3, 13.1 Hz, H-3α), 2.84 (1H, *dd*, *J* = 17.3, 3.1 Hz, H-3β), 12.82 (1H, *s*, 5-OH), 6.13 (1H, *s*, H-8), 3.85 (2H, *s*, H-7′′), 7.43-7.44 (2H, *m*, H-2′/6′), 7.43-7.44 (2H, *m*, H-3′/5′), 7.43-7.44 (1H, *m*, H-4′), 6.86 (1H, *dd*, *J* = 8.1, 1.0 Hz, H-3′′), 7.10 (1H, *td*, *J* = 8.0, 1.7 Hz, H-4′′), 6.82 (1H, *td*, *J* = 7.5, 1.1 Hz, H-5′′), 3.92 (3H, *s*, 7-OCH_3); ^{13}C-NMR ($CDCl_3$, 25 ^oC) δ (200 MHz, ppm): 79.7 (C-2), 43.3 (C-3), 196.3 (C-4), 103.0 (C-4a), 159.0 (C-5), 109.1 (C-6), 165.4 (C-7), 92.1 (C-8), 162.0 (C-8a), 138.2 (C-1′), 126.3 (C-2′/6′), 129.1 (C-3′/5′), 129.2 (C-4′), 125.9 (C-1′′), 154.7 (C-2′′), 116.6 (C-3′′), 128.0 (C-4′′), 120.1 (C-5′′), 131.8 (C-6′′), 22.8 (C-7"), 56.3 (-OCH_3).

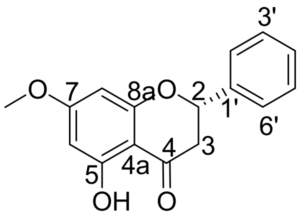

5-Hydroxy-7-methoxyflavanone (Pinostrobin, ***6****)*: [4] Yellow powder. [$[\alpha]_D^{20}$ - 37.5 (c, 1.3, MeOH), UV (MeOH)$_{\lambda max}$ 275nm; CD (MeOH, λnm ($\Delta\varepsilon$; $M^{-1}cm^{-1}$): (+13.45)$_{356}$; (- 2.33)$_{335}$; (- 6.85)$_{257}$; (+ 3.03)$_{246}$; IR (KBr) ν_{max} 3429, 1640, 1423, 1265, 738 cm^{-1}; LC-MS *m/z* 271.6 $[M+H]^+$, 251.6, 229.5, 187.4, 167.3, 149.6, 131.1, 144.4, 102.4, 74.1, 55.5; ^{1}H-NMR ($CDCl_3$, 25 °C) δ (800 MHz, ppm): 5.43 (1H, *dd*, *J* = 13.2, 3.0 Hz, H-2), 3.09 (1H, *dd*, *J* = 17.2, 13.2 Hz, H-3α), 2.83 (1H, *dd*, *J* = 17.1, 3.0 Hz, H-3β), 6.07 (1H, *d*, *J* = 2.3 Hz, H-6), 6.09 (1H, *d*, *J* = 2.3 Hz, H-8), 7.38-7.47 (2H, *m*, H-2′/6′), 7.38-7.47 (2H, *m*, H-3′/5′), 7.38-7.47 (1H, *m*, H-4′), 3.82 (3H, *s*, 7-OCH_3), 12.02 (1H, *s*, 5-OH); ^{13}C-NMR ($CDCl_3$, 25 °C) δ (200 MHz, ppm): 79.4 (C-2), 43.6 (C-3), 195.9 (C-4), 103.3 (C-4a), 164.3 (C-5), 95.3 (C-6), 168.1 (C-7), 94.4 (C-8), 162.9 (C-8a), 138.5 (C-1′), 126.3 (C-2′/6′), 129.0 (C-3′/5′), 129.0 (C-4′), 55.9 (-OCH_3).

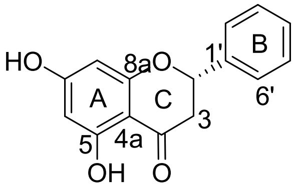

Pinocembrin (7): [5] White powder. mp 205 – 208 °C; $[\alpha]_D^{20}$ - 20.8 (c, 1.5, MeOH), UV (MeOH)$_{\lambda max}$ 295, 210 nm; IR (KBr) ν_{max} 3430, 1640, 1265, 740 cm^{-1}; LC-MS *m/z* 257.6 [M+H]$^+$, 237.1, 233.1, 223.5, 207.4, 183.1, 166.3, 151.3, 149.5, 145.2, 129.0; ^{1}H-NMR ($CDCl_3$, 25 ^{o}C) δ (800 MHz, ppm): 12.04 (1H, *s*, H-5-OH), 7.45 (4H, *m*, H-2′/6′ and H-3′/5′), 7.40 (1H, *m*, H-4′), 6.00 (2H, *s*, H-6 and H-8), 5.43 (1H, *dd*, *J* = 13.1 and 3.1 Hz, H-2), 3.09 (1H, *dd*, *J* = 17.1, 13.1, H-3α), 2.83 (1H, *dd*, *J* = 17.1, 3.1 Hz, 3β); ^{13}C-NMR ($CDCl_3$, 25 ^{o}C) δ (200 MHz, ppm): 79.4 (C-2), 43.5 (C-3), 195.9 (C-4), 103.4 (C-4a), 164.5 (C-5), 96.9 (C-6), 164.5 (C-7), 95.6 (C-8), 163.3 (C-8a), 138.4 (C-1′), 129.1 (C-4′), 129.0 (C-3′/5′), 126.3 (C-2′/6′).

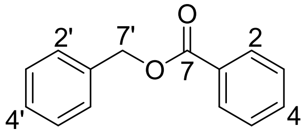

Benzylbenzoate (8): [6] Colourless oil. UV (MeOH)$_{\lambda max}$ 225nm; IR (KBr) ν_{max} 3425, 3056, 1641, 1423, 1265, 894, 739 cm^{-1}; LC-MS *m/z* 213.1 [M+H]$^+$, 189.4, 181.1, 175.4, 160.3, 149.4, 137.4, 129.0, 121.0, 114.2, 122.3, 98.9, 91.3,; ^{1}H-NMR ($CDCl_3$, 25 ^{o}C) δ (800 MHz, ppm): 8.08 (2H, *d*, *J* = 8.1 Hz, H-2/6), 7.45 (2H, *m*, H-3/5), 7.56 (1H, *m*, H-4), 7.46 (2H, *m*, H-2′/6′), 7.39 (2H, *t*, *J* = 7.4 Hz, H-3′/5′), 7.40 (1H, *d*, *J* = 7.4 Hz, H-4′), 5.37 (2H, *s*, H-7′); ^{13}C-NMR ($CDCl_3$, 25 ^{o}C) δ (200 MHz, ppm): 132.7 (C-1), 132.3 (C-2/6), 131.0 (C-3/5), 135.7 (C-4), 131.2 (C-2′/6′), 130.8 (C-3′/5′). 130.9 (C-4′), 138.7 (C-1′), 169.1 (C- 7), 69.3 (C- 7′).

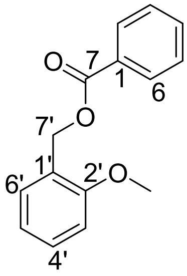

2-Methoxybenzylbenzoate (***9***): [6] Colourless oil. UV (MeOH)$_{\lambda max}$ 225, 275nm; LC-MS *m/z* 265.2 [M+Na]$^+$, 243.7, 241.4, 218.5, 196.2, 175.4, 149.5, 129.0, 125.2, 121.5, 120.7, 114.4, 98.4, 91.2, 79.2, 60.3; 8.11; ^{1}H-NMR ($CDCl_3$, 25 °C) δ (800 MHz, ppm): (2H, *dd*, *J* = 8.1, 0.9 Hz, H-2/6), 7.55 (1H, *m*, H-1), 7.44 (2H, *m*, H-3′/5′), 7.42 (1H, *m*, H-4′), 7.33 (1H, *m*, H-6′), 6.97 (1H, *t*, *J* = 7.4 Hz, H-5′), 6.92 (1H, *d*, *J* = 8.2 Hz, H-3′), 5.42 (2H, *s*, H-7′), 3.86 (3H, *s*, -OCH_3); ^{13}C-NMR ($CDCl_3$, 25 °C) δ (200 MHz, ppm): 124.9 (C-1′), 130.6 (C-1), 157.7 (C-2′), 129.9 (C-2/6), 133.0 (C-4), 128.5 (C-3/5), 166.7 (C-7), 124.9 (C-1'), 157.7 (C-2'), 110.6 (C-3′), 129.6 (C-4′), 120.6 (C-5′), 129.6 (C-6′), 62.3 (C-7′), 55.6 (-OCH_3).

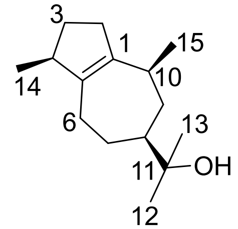

Guaiol (***10***): [7] White crystals. mp 91 – 92 °C; $[\alpha]_D^{20}$ + 97.5 (c, 0.15, $CHCl_3$); UV ($CHCl_3$)$_{\lambda max}$ 205 nm; CD (MeOH, λnm (Δε; $M^{-1}cm^{-1}$): (- 7.31)$_{242}$; (+ 1.26)$_{210}$; IR (KBr) ν_{max} 3429, 1641,1423, 1265, 739 cm^{-1}; LC-MS *m/z* 245.7 [M+Na]$^+$, 228.7, 223.5, 205.4, 163.7, 150.2, 149.5, 135.6, 114.2, 109.4, 107.2, 95.4, 93.2, 81.4, 72.2. ^{1}H-NMR ($CDCl_3$, 25 °C) δ (800 MHz,

ppm): 2.53 (1H, *m*, H-4), 2.43 (1H, *m*, C-2a), 2.30 (1H, *m*, H-10), 2.15 (1H, *d*, *J* = 15.7 Hz, H-6a),2.12 (1H, *m*, H-2b), 1.95 (2H, *m*, H-6b and H-3a), 1.81 (1H, *dddd), J* = 13.0, 9.6, 4.0, , 1.7 Hz, H-7a), 1.72 (1H, *m*, J = 13.7, 9.4, 7.6, 3.4 Hz, H-9a), 1.56 (2H, *m*, H-9b and H-8), 1.45 (1H, *dddd*, *J* = 13.0, 10.6, 7.5, 3.4 Hz, H-7b), 1.29 (1H, *ddd), J* = 12.6, 9.0, 5.0, Hz, H-3b), 1.20 (1H, *s*, OH), 1.19 (3H, *s*, CH_3-12), 1.16 (3H, *s*, CH_3-13), 0.99 (3H, *d*, J = 7.2 Hz, CH_3-15), 0.95 (3H, *d*, J = 6.9 Hz, CH_3-14); ^{13}C-NMR ($CDCl_3$, 25 ^oC) δ (200 MHz, ppm): 140.2 (C-1), 139.0 (C-5), 73.7 (C-11), 49.8 (C-8), 46.5 (C-4), 35.5 (C-2), 33.9 (C-9), 33.8 (C-10), 31.1 (C-3), 28.0 (C-6), 27.6 (C-12), 27.5 (C-7), 26.2 (C-13), 20.1 (C-14), 19.9 (C-15).

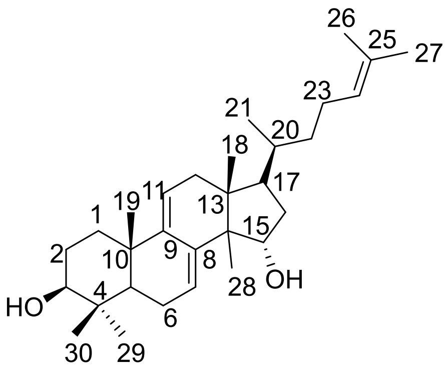

*Polycarpol (**11**)*: [2, 5, 8] White crystals. mp 184 – 185 °C; [$[\alpha]_D^{20}$ + 97.5 (c, 5.8, CH_2Cl_2), UV (CH_2Cl_2)$_{\lambda max}$ 245nm; CD (MeOH, λnm (Δε; $M^{-1}cm^{-1}$): (- 7.67)$_{290}$; (+ 3.61)$_{212}$; IR (KBr) ν_{max} 3418, 3055, 1640, 1423, 1265, 895, 739 cm^{-1}; LC-MS *m/z* 463.5 $[M+Na]^+$, 428.9, 416.6, 377.4, 371.6, 343.2, 320.9, 302.5, 259.4, 251.4, 227.1, 175.5, 149.6, 142.0, 125.1, 123.3, 98.1, 83.3, 60.3; ^{1}H-NMR ($CDCl_3$, 25 ^oC) δ (800 MHz, ppm): 1.98 (1H, *m*, H-1α) 1.43 (1H, *m*, H-1β), 1.72 (1H, *m*, H-2α), 1.65 (1H, *m*, H-2β), 3.24 (1H, *dd*, *J* = 11.6, 3.9 Hz, H-3α), 1.45 (1H, *dd*, *J*

= 13.3, 3.9 Hz, H-5), 2.16 (1H, *m*, H-6α), 2.07 (1H, *m*, H-6β), 5.85 (1H, *d*, *J* = 6.2 Hz, H-7), 5.30 (1H, *d*, *J* = 6.2 Hz, H-11), 2.29 (1H, *m*, H-12α), 2.05 (1H, *m*, H-12β), 4.27 (1H, *m*, H-15), 1.96 (1H, *m*, H-16α), 1.72 (1H, *m*, 16β), 1.35 (1H, *m*, H-17), 0.61 (3H, *s*, 18-CH_3), 0.93 (3H, *s*, 19-CH_3), 1.86 (1H, *m*, H-20), 0.88 (3H, *s*, 21-CH_3), 1.35 (1H, *m*, H-22α), 1.03 (1H, *m*, H-22β), 2.03 (1H, *m*, H-23), 1.85 (1H, *m*, H-23), 5.08 (1H, *t*, *J* = 6.9 Hz, H-24), 1.60 (3H, *s*, H-26), 1.68 (3H, *s*, H-27), 0.88 (3H, *s*, H-28), 0.98 (3H, *s*, H-29), 1.00 (3H, *s*, H-30); ^{13}C-NMR ($CDCl_3$, 25 ^{o}C) δ (200 MHz, ppm): 35.9 (C-1), 27.9 (C-2), 79.0 (C-3), 38.8 (C-4), 49.1 (C-5), 23.1 (C-6), 121.4 (C-7), 141.0 (C-8), 146.2 (C-9), 37.6 (C-10), 116.2 (C-11), 38.6 (C-12), 44.5 (C-13), 51.9 (C-14), 74.9 (C-15), 40.3 (C-16), 49.0 (C-17), 16.1 (C-18), 23.0 (C-19), 36.4 (C-20), 18.5 (C-21), 35.9 (C-22), 25.0 (C-23), 125.1 (C-24), 131.3 (C-25), 25.9 (C-26), 17.8 (C-27), 17.3 (C-28), 28.3 (C-29), 16.0 (C-30).

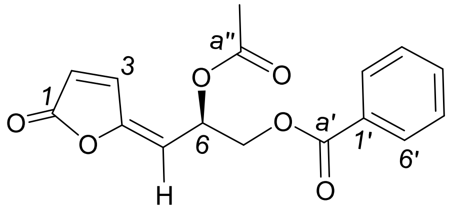

(E)-Acetylmelodorinol (***12***): [5] Colourless oil. [$[\alpha]_D^{20}$ – 2.8 (c, 1.1, MeOH), UV (MeOH)$_{\lambda max}$ 265, 225 nm; CD (MeOH, *λ*nm (Δε; $M^{-1}cm^{-1}$): (- 0.99)$_{329}$; (+ 1.31)$_{311}$; (- 1.56)$_{301;}$ (+1.34)$_{281}$; IR (KBr) ν_{max} 3430, 2082, 1640, 1265, 739 cm^{-1}; LC-MS *m*/*z* 303.4 $[M+H]^+$ 291.1, 274.6, 273.4, 251.4, 231.3, 175.8, 149.5, 129.0, 109.5, 105.1, 91.3, 81.4, 79.1, 73.9, 60.1; ^{1}H-NMR ($CDCl_3$, 25 ^{o}C) δ (800 MHz, ppm): 6.34 (1H, *d*, *J* = 5.7 Hz, H-2), 7.90 (1H, *d*, J = *5.7* Hz, H-3), 5.72

(1H, *dd*, *J* = 10.0, 1.1 Hz, H-5), 5.98 (1H, *ddd*, *J* = 10.8, 67, 4.2 Hz, H-6), 4.53 (1H, *dd*, *J* = 11.8, 4.2 Hz, H-7α), 4.47 (1H, *dd*, *J* = 11.6, 6.7 Hz, H-7β), 8.01 (2H, *dd*, *J* = 8.1, 1.1 Hz, H-2′/6′), 7.46 (2H, *t*, *J* = 7.8, 7.8 Hz, H-3′/5′), 7.60 (1H, *m*, H-4′), 2.10 (3H, *s*, -CH_3), ^{13}C-NMR ($CDCl_3$, 25 ^{o}C) δ (200 MHz, ppm): 168.7 (C-1), 122.6 (C-2), 140.2 (C-3), 153.5 (C-4), 107.5, (C-5), 66.8 (C-6), 65.1 (C-7), 129.3 (C-1′), 129.7 (C-2′/6′), 128.6 (C-3′/5′), 133.5 (C-4′), 166.0 (C-α′), 170.1 (C-α″).

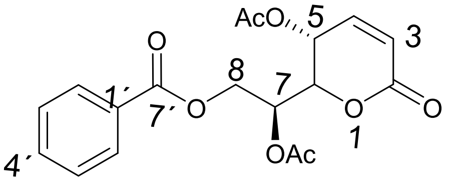

*Cleistenolide (**13**)*: [2, 5] White needles. Green upon spraying with anisaldehyde reagent; mp 128-130 ^{o}C; IR 2965 cm^{-1}, 1749.72 cm^{-1}, 1601.20 cm^{-1}; ^{1}H-NMR ($CDCl_3$, 25 ^{o}C) δ (800 MHz, ppm): 6.31 (*d*, 9.6, H-3), 7.02 (*dd*, 9.6, 6.4, H-4), 5.44 (*dd*, 6.4, 3.2, H-5), 4.82 (*dd*, 10.4, 2.4, H-6), 5.52 (*ddd*,10.4, 4.4, 2.4, H-7), 4.56 (*dd*, 12.8, 4.8 H-8α), 4.95 (*dd*, 12.8, 2.4, H-8β), 2.06 (H-11), 2.11 (*s*, H-13), 8.04 (*dd*, 8.8, 0.8, H-2′), 7.48 (*dd*, 8.0, 8.0, H-3′), 7.60 (*t*, 8.0, H-4′) 7.48, (*dd* (8.0, 8.0, H-5′), 8.04 (*dd* 8.8, 0.8 H-6′); ^{13}C-NMR ($CDCl_3$, 25 ^{o}C) δ (200 MHz, ppm): 161.2 (C-2), 125.5 (C-3), 139.9 (C-4), 75.6 (C-5), 170.0 (5-O$\underline{C}$OCH_3), 20.6 (5-$\underline{C}H_3$COO), 59.9 (C-6), 67.8 (C-7), 169.6 (7-O$\underline{C}$OCH_3), 20.8 (7-$\underline{C}H_3$COO) 62.2 (C-8), 166.9 (7′-O$\underline{C}$OPh), 129.7 (C-1′), 129.8 (C-2′/6′), 128.6 (C-3′/5′), 133.4 (C-4′)

3. X-Ray crystallography for 7-methoxyisochamanetin (5) and guaiol (10)

X-Ray data for single crystals of **5** and **10** were collected on an Agilent SuperNova Dual source diffractometer, equipped with an Atlas detector, at T = 120.0 K for **5** and T = 123.0 K for **10** using mirror-monochromatized Cu-Kα radiation (λ = 1.54184 Å). The data collection and reduction were performed using the program *CrysAlisPro*, [9] and the intensities were corrected using Gaussian face index absorption correction method [9] for **5** and **10**. The structure was solved by Intrinsic Phasing method with SHELXT [10] and refined by full-matrix least-squares using SHELXL-2015 [11] within *OLEX2* package. [12] All non-hydrogen atoms were refined anisotropically, and all the hydrogen atoms were refined using riding models with U_{eq}(H) of 1.5U_{eq}(parent) for terminal groups and 1.2 U_{eq}(parent) for non-terminal groups.

Compound **5**: 0.335×0.085×0.066 mm, $C_{23}H_{20}O_5$, M = 376.39, orthorhombic, space group $P2_12_12_1$, a = 5.27836(4) Å, b = 15.81290(14) Å, c = 21.27803(16) Å, α = 90°, β = 90°, γ = 90°, V = 1776.00(2) Å^3, Z = 4, ϱ = 1.408 g cm^{-3}, μ = 0.811 mm^{-1}, F(000) = 792, 25733 reflections (θ_{max} = 66.746°) measured (3154 unique, R_{int} = 0.0338, completeness = 99.89%), Final R indices ($I > 2\sigma(I)$): R_1= 0.0275, wR_2 = 0.0709, R indices (all data): R_1= 0.0279, wR_2 = 0.0713. GoF = 1.081 for 256 parameters and 0 restraints, largest diff. peak and hole 0.140/–0.186 eÅ^{-3}. Absolute structure parameter x = -0.07(5). CCDC number - 1937081 contains the supplementary data for this structure.

Compound **10**: 0.188×0.081×0.075 mm, $C_{15}H_{26}O$, M = 222.36, trigonal, space group $P3_2$, a = 13.02112(10) Å, b = 13.02112(10) Å, c = 7.05780(5) Å, α = 90°, β = 90°, γ = 120°, V = 1036.327(18) Å^3, Z = 3, ϱ = 1.069 g cm^{-3}, μ = 0.483 mm^{-1}, F(000) = 372, 16437 reflections (θ_{max} = 76.545°) measured (2884 unique, R_{int} = 0.0254, completeness = 99.99%), Final R indices ($I > 2\sigma(I)$): R_1= 0.0370, wR_2 = 0.0986, R indices (all data): R_1= 0.0371, wR_2 = 0.0988. GOF = 1.089 for 150 parameters and 1 restraint, largest diff. peak and hole 0.334/–0.290 eÅ^{-3}. Absolute structure parameter x = 0.01(6). CCDC number - 1937082 contains the supplementary data for this structure.

4. References

1. Achenbach, H.; Höhn, M.; Waibel, R.; Nkunya, M.H.; Jonker, S.A.; Muhie, S. Oxygenated pyrenes, their potential biosynthetic precursor and benzylated dihydroflavones from two African Uvaria species. *Phytochem.* **1997**, *44*, 359-364.
2. Pereira, F.; Madureira, A.M.; Sancha, S.; Mulhovo, S.; Luo, X.; Duarte, A.; Ferreira, M.-J.U. Cleistochlamys kirkii chemical constituents: Antibacterial activity and synergistic effects against resistant Staphylococcus aureus strains. *J. Etnopharm.* **2016**, *178*, 180-187.
3. Lasswell Jr, W.L.; Hufford, C.D. Cytotoxic C-benzylated flavonoids from Uvaria chamae. *J. Org. Chem.* **1977**, *42*, 1295-1302.
4. Ching, A.Y.L.; Wah, T.S.; Sukari, M.A.; Lian, G.E.C.; Rahmani, M.; Khalid, K. Characterization of flavonoid derivatives from Boesenbergia rotunda (L.). *Malaysian J. Anal. Sci.* **2007**, *11*, 154-159.
5. Samwel, S.; Mdachi, S.J.M.; Nkunya, M.H.H.; Irungu, B.N.; Moshi, M.J.; B., M.; B.S., L. Cleistenolide and cleistodienol: Novel bioactive constituents of Cleistochlamys kirkii. *Nat. Prod. Commun.* **2007**, *2*, 737-741.
6. Kodpinid, M.; Thebtaranonth, C.; Thebtaranonth, Y. Benzyl benzoates and o-hydroxybenzyl flavanones from Uvaria ferruginea. *Phytochem.* 1985, *24*, 3071-3072.
7. Liu, T.; Wang, C.-J.; Xie, H.-Q.; Mu, Q. Guaiol - a naturally occurring insecticidal sesquiterpene. *Nat Prod. Commun.* **2013**, *8*, 1353-1354.
8. Jung, J.; Pummangura, S.; Chaichantipyuth, C.; Patarapanich, C.; Fanwick, P.; Chang, C.-J.; McLaughlin, J. New bioactive heptenes from Melodorum fruticosum (Annonaceae). *Tetrahedron* **1990**, *46*, 5043-5054.
9. Diffraction, R.O. *CrysAlisPro* Version 38.46; 2018.
10. Sheldrick, G.M. SHELXT - Integrated space-group and crystal-structure determination. *Acta Crystallogr A* **2015**, *71*, 3-8.
11. Sheldrick, G.M. Crystal structure refinement with SHELXL. *Acta Crystallogr C* **2015**, *71*, 3-8.
12. Dolomanov, O.V.; Bourhis, L.J.; Gildea, R.J.; Howard, J.A.K.; Puschmann, H. OLEX2: a complete structure solution, refinement and analysis program. *J Appl Crystallogr* **2009**, *42*, 339-341.
